# Face Masks for All and All for Face Masks in the COVID-19 Pandemic: Community Level Production to Face the Global Shortage and Shorten the Epidemic

**DOI:** 10.1017/dmp.2020.207

**Published:** 2020-06-24

**Authors:** Eduardo Missoni, Benedetta Armocida, Beatrice Formenti

**Affiliations:** Saluteglobale.it, Associazione di Promozione Sociale, Brescia, Italy; Centre for Research on Health and Social Care Management, Bocconi University, Milano, Italy

**Keywords:** COVID-19, homemade masks, medical masks, pandemic prevention, personal protection equipment

## Abstract

The current coronavirus disease 2019 (COVID-19) pandemic caused a global shortage of medical masks, leaving most exposed health personnel without appropriate protection.

Since the beginning of the outbreak, the World Health Organization WHO) has revised several times the recommendations on general use of facemasks. Until recently, WHO recommended to limit the use of facemasks to symptomatic people and advised against off-standard solutions. Moreover, recommendations differ among and within countries, causing public confusion and individual initiative.

There is wide consensus that universal appropriate use of masks may contribute both to contain the epidemic and to reduce the burden on national procurement, if a community production approach is followed. Especially in low-middle income countries, due to the scarce capacity of national industrial production or import, the use of masks produced at community level may become the only viable option. For the purpose ad hoc guidelines will be needed.

Current knowledge and experience call for further and updated review of global and national guidelines to provide clear and consistent criteria to ensure the widest availability and appropriate use of facial protection, bearing in mind populations in socio-economic disadvantaged settings.

The current coronavirus disease 2019 (COVID-19) pandemic caused a dramatic global shortage of personal protection equipment (PPE), including medical masks, often leaving most exposed health personnel without appropriate protection.^1^

Latest 2019 pre-COVID-19 World Health Organization (WHO) guidelines for mitigating the risk and impact of epidemic and pandemic influenza would “conditionally recommend” face masks worn by asymptomatic people in severe epidemics or pandemics. Although in the absence of evidence, the guidelines explicitly admitted a mechanism of plausibility for the potential effectiveness of this measure in reducing transmission in the community.^2^

Since the beginning of the COVID-19 outbreak, WHO has revised several times the recommendations on general use of face masks. Probably with the intention to ensure essential supply, at the early stage of the pandemic WHO advised the general public to limit the use of medical masks to symptomatic people and clearly advised against off-standard solutions (ie, cloth masks) “under any circumstance.” However, with contradiction, adding that “masks might be worn in some countries according to local cultural habits”^3^ where often masks are indeed reusable and made of cloth. These inconsistencies were considered a possible cause of public confusion.^4,5^ In April, new guidelines softened WHO’s position toward face masks use in the community. The new document stated that “wearing a medical mask is one of the prevention measures to limit spread of certain respiratory diseases, including 2019-nCoV,” adding though that “the wide use of masks by healthy people in the community setting is not supported by current evidence and carries uncertainties and critical risks.” Moreover the new guidelines advised decision-makers to apply a risk-based approach, highlighting that there is no current evidence to make a recommendation for or against nonmedical masks use in the community setting; however, clarifying that no criticism was moved toward countries who suggest wearing masks, and remarking the importance of advice about how to wear and dispose of them.^6^ In June, after almost 3 months from the announcement of COVID-19 pandemic and 6 months from the start of the epidemic, WHO advised governments to encourage the general public to use face masks, both medical and nonmedical, as part of a comprehensive approach to limit severe acute respiratory syndrome coronavirus-2 (SARS-CoV-2) transmission. Although stating that the widespread use of masks in healthy people is not yet supported by high quality or direct scientific evidence, the potential benefits and harms should be considered.^7^

During the peak of the pandemic, WHO recommendations contrasted with those of other public health agencies. In light of the evidence that a significant portion of asymptomatic and presymptomatic individuals can transmit the virus to others before showing symptoms, since the beginning CDC suggested “wearing cloth face coverings in public settings where other social distancing measures are difficult to maintain.”^8^ The European Centre for Disease Prevention and Control (ECDC) has followed a similar recommendation.^9^ Additionally, recommendations differ also among and within countries.^5^ When the epidemic was peaking, the Italian government strictly followed the initial WHO recommendations, while the Lombardia Region made the use of facemasks compulsory in public, an approach subsequently adopted by the central government, but only where distancing could not be ensured.^10^ China requested to wear masks in public,^11^ and the Czech Republic made it compulsory to wear any mouth- and nose-covering apparel when in public, launching a nationwide DoItYourself campaign.^12^

This fragmented scenario has generated confusion and doubts about the universal use of facemasks.

We understand the earliest WHO’s concerns related to costs, procurement burden, false sense of security, or reduced effectiveness due to incorrect use of wearing masks. We are also aware of some medical warnings.^13^ However, we assert that a well-oriented community-focused approach is the most adequate response to those concerns. Along this line, we argue that international guidelines have to be constantly updated and revised considering also (1) the available and progressive evidence of the effectiveness universal use of face masks in preventing the spread of the coronavirus infection; and (2) the benefits of involving communities in the mask-making enterprise, if clear instructions are provided, suggesting appropriate technical specifications and to prevent misuse.

While PPE should ideally respond to recognized safety and quality standards, the current pandemic is by no means an ideal situation, and even in high-income countries, such as the United States of America (USA) and Italy, the market is dramatically failing in providing on-standard face protection including to the most exposed personnel. Under these circumstances, community level production may be the solution to protect the general public and release resources for the health-care personnel. With limited exceptions, the situation in low-middle income countries (LMICs) will inevitably be worse. Due to the scarce capacity of national production or import, it is highly unlikely that they will be able to provide enough certified disposable masks to all their population. To overcome the global shortage, especially in LMICs, a viable option would be the production of masks at community and/or domestic level.^14^ This solution will be most effective if accompanied by appropriate instructions and training, as well as the indication of the best possible technical solution according to the local setting.

## THE RATIONALE BEHIND “FACE MASKS FOR ALL”

Evidence suggests that facemasks play a pivotal role in the prevention and control of infectious respiratory disease transmission,^15,16^ and nose-mouth barriers reduce—to an extent limited according to their technical characteristics (for example, cloth masks have proved to be less effective than medical masks)^17^—the exposure of healthy people to infection, and to a higher extent the capacity of infected people to spread infection by means of droplets.^18^

Furthermore, it has been reported that SARS-CoV-2 can be transmitted by mildly ill or even asymptomatic people^19,20^; thus, limiting the use of face masks to symptomatic people may allow wide uncontrolled spread of the virus. The importance of adequate protective barriers, for both symptomatic and asymptomatic people becomes self-evident, suggesting that, during epidemics, nose and mouth protection should be universal in all situations where appropriate distancing is not possible or unpredictable, especially in closed spaces. Some of the countries that seem to have been more successful in the control of the epidemic enacted policies toward that goal.

In South Korea, for example, health officials have urged all citizens to wear filtered masks, with the Seoul government seizing near total control of face-mask production, distribution, and sales.^21^ A similar approach was implemented by Taiwan, where the government, recognizing the importance of ensuring an adequate supply of medical equipment, first, stopped exports of surgical face masks and requested local companies to increase production; second, took control of face mask distribution from the private sector, to ensuring no hoarding of supplies or exploitative pricing; and third, established a purchasing policy.^22^ This policy allows every Taiwanese to buy a certain amount of adult and children’s masks per week from pharmacies and clinics for a controlled price.

## THE RATIONALE BEHIND “ALL FOR FACE MASKS”

If we agree on the usefulness of appropriate universal use of masks to limit the spread of the virus by means of droplets, the new challenge becomes providing masks to everybody, ensuring their effective and safe use, and making the production as sustainable as possible, which suggests local availability, affordability, and reusability.

Regarding availability, where capability for industrial production of high standard PPE exists and can be increased to the needs, this would possibly be the best choice. Import instead may not be an intelligent option for granting an effective national pandemic response. According to WHO modeling, an estimated 89 million medical masks are required for the COVID-19 response each month. To meet rising demand, manufacturing should increase globally by 40%.^1^ However, the pandemic also challenged the functioning of the global market, with producing countries banning their exports to meet the foreseeable increase in national demand. When the epidemic was peaking, even China—the world’s main producer—asked for international support for medical masks and other PPEs. In Italy, despite the initial official recommendation of the Ministry of Health, there was a general rush to buy all possible kinds of medical masks and respirators, enormously increasing costs and hindering supply to first-line workers.

Limiting use to disposable PPE will come with an additional burden to national systems, due to the need to dispose of it adequately. The Chinese experience highlights this problem: at the peak of the COVID-19 outbreak, hospitals in Wuhan increased the medical waste from PPE by 6 times—to nearly 270 tons/d.^23^

With the need increasing, in social media homemade solutions flourished, mostly without any evidence about their effectiveness, but creativity and commonsense. As this movement cannot be stopped—people are mainly moved by their individualistic desire of personal protection—then it should be managed.

Local community production may be a viable option and possibly the most appropriate one in socially and economically disadvantaged settings. However, best possible solutions need to be identified and promoted according to effectiveness, safety, availability, affordability, and sustainability criteria.

Despite WHO initial advice against cloth masks^3^ and although cloth masks have lower filtration capacity compared with disposable medical masks, evidence suggests that any type of mask use is likely to decrease viral exposure and infection risk on a population level, despite imperfect fit and imperfect adherence, still offering some level of protection.^18,24^

In addition to cloth, some combination of different materials easily available at community/domestic level could be more effective, with protection increasing according to the fineness of the fabric and the number of layers.^14^ In a study, vacuum cleaner bags and double layer tea towels proved to have a filtration power very near to that of surgical masks, although due to a too high a pressure drop causing insufficient breathability, these materials could not be considered good mask materials. T-shirts or pillowcases made of 100% cotton are suggested as the most suitable homemade mask material.^24^

The standard for industrially produced medical masks is mainly based on nonwoven material. Widely commercialized domestic tools are also made of nonwoven material (such as cleaning towels or cotton pads), giving the opportunity to widely test them for the domestic production of protective masks.

A recent systematic review and meta-analysis concludes that policy-makers across the globe should guarantee and protect equal access to face masks for every social group. Government interventions should facilitate and increase the production capacity of facemasks, enabling widespread use by the general public and for those with limited access to facemasks.^16^

The local production of face masks comes with several pros and cons, these are presented in Table 1. Among others the diffuse production and universal use of masks may improve the perception of public health risks and strengthen the public sense of both personal and community control.^25^

**TABLE 1:** Pros and Cons of Local Production of Face Masks

Pros	Cons
Wide and timely availability at local level, prevents shortages of masks for professional use.	Lack of standardization and quality control; diversity of materials used and technical characteristics (filterability, breathability, fitness)
Affordable, easy, and cheap manufacture	Limited choice of materials in rural areas and poor settings
Environmental sustainability (depending on reusability and materials used)	Not adequate to supply caregivers and people at high risk of exposure
Independent from global market dynamics	
Community empowerment	

The promotion of community production and universal use of facemasks, should be supported with ad hoc guidelines. Their development may need further interdisciplinary research. Indeed, it should not be limited to technical aspects (safety and biological effectiveness of materials and models, decontamination procedures), but include epidemiological (health outcomes), social and anthropological aspects (correct and effective use), impact on local economy, and the characteristics of effective communication strategies.

In any case, to avoid possible increased risk of transmission associated with the incorrect use and disposal or maintenance and decontamination of face masks, appropriate and widely diffused instructions should necessarily accompany the promotion of masks use and production^3^ and always insist on the fundamental need to combine the use of masks with the prioritarian hand hygiene and other infection prevention and control (IPC) measures.

## CONCLUSIONS

To conclude, during epidemics, medical masks, and if not available, appropriate homemade masks, should be universally worn when keeping physical distance is not possible. Together with other IPC measures, this practice will increase community protection and empowerment. Homemade masks additionally contribute to freeing-up resources and more effective equipment that should be absolutely reserved to health-care and other workers at highest risk. Further research may help to identify even more effective, safe, available, affordable, and sustainable community level solutions. Nevertheless, current situation, knowledge, and experience call for further and updated review of global and national guidelines and recommendations to provide, together with other IPC measures, clear and consistent criteria to ensure the widest availability and appropriate use of facial protection, bearing especially in mind most vulnerable populations in socio-economic disadvantaged settings. We believe that the universal appropriate use of effective reusable masks and their cooperative production may prompt the sense of personal and collective responsibility in building a resilient society where nobody should be left behind.
