# Face Masks for All and All for Face Masks in the COVID-19 Pandemic: Community Level Production to Face the Global Shortage and Shorten the Epidemic – Corrigendum

**DOI:** 10.1017/dmp.2021.17

**Published:** 2021-02

**Authors:** Eduardo Missoni, Benedetta Armocida, Beatrice Formenti

**Keywords:** COVID-19, homemade masks, medical masks, pandemic prevention, personal protection equipment, corrigendum

The original publication of this article^1^ listed only one of Dr. Benedetta Armocida’s affiliations. Her primary affiliation is Institute for Maternal and Child Health, IRCCS “Burlo Garofolo”, Trieste, Italy.
